# Heat shock protein 47 promotes tumor survival and therapy resistance by modulating AKT signaling *via* PHLPP1 in colorectal cancer

**DOI:** 10.20892/j.issn.2095-3941.2019.0261

**Published:** 2020-05-15

**Authors:** Yijye Chern, Peter Zhang, Hyelim Ju, Isabella T. Tai

**Affiliations:** ^1^Division of Gastroenterology, Department of Medicine, University of British Columbia, Vancouver V6T 1Z4, Canada; ^2^Michael Smith Genome Sciences Center, British Columbia Cancer Agency, Vancouver V5Z 4S6, Canada

**Keywords:** HSP47, AKT, PHLPP1, colorectal cancer, resistanc

## Abstract

**Objective:** Heat shock protein 47 (HSP47) is a collagen-specific molecular chaperone that facilitates collagen maturation. Its role in cancer remains largely unknown. In this study, we investigated the roles of HSP47 in colorectal cancer (CRC) and therapy resistance.

**Methods:** Expression of HSP47 in CRC tissues was examined (1) in paired human CRC/adjacent normal tissues, using real time quantitative reverse transcription polymerase chain reaction (qRT-PCR), The Cancer Genome Atlas (TCGA) database, and 22 independent microarray databases (curated CRC). *In vitro* studies on several CRC cell lines (HCT116, RKO and CCL228) with modulated HSP47 expression were conducted to assess cell viability and apoptosis (TUNEL assay and caspase-3/-7) during exposure to chemotherapy. AKT signaling and co-immunoprecipitation studies were performed to examine HSP47 and PHLPP1 interaction. *In vivo* studies using tumor xenografts were conducted to assess the effects of HSP47 modulation on tumor growth and therapy response.

**Results:** HSP47 was upregulated in CRC and was associated with poor prognosis in individuals with CRC. *In vitro*, HSP47 overexpression supported the survival of CRC cells, whereas its knockdown sensitized cells to 5-fluorouracil (5-FU). HSP47 promoted survival by inhibiting apoptosis, enhancing AKT phosphorylation, and decreasing expression of the AKT-specific phosphatase PHLPP1 when cells were exposed to chemotherapy. These effects were partly results of the interaction between HSP47 and PHLPP1, which decreased PHLPP1 stability and led to more persistent AKT activity. *In vivo*, HSP47 supported tumor growth despite 5-FU treatment.

**Conclusions:** HSP47 supports the growth of CRC tumors and suppresses the efficacy of chemotherapy *via* modulation of AKT signaling.

## Introduction

Colorectal cancer (CRC) is among the three most commonly diagnosed cancers worldwide. The 5-year survival rate of patients with CRC diagnosed with distant metastasis is only 14% in the United States^1^. Approximately 25% of individuals present with distant tumors at the initial diagnosis, and almost half of all patients develop metastases within 5 years^2^, thus indicating an urgent need for the development of effective therapy for metastatic CRC (mCRC). The primary cause of treatment failure in patients with mCRC is the emergence of resistance toward chemotherapy. Therefore, understanding the mechanisms underlying drug resistance in CRC is essential for developing successful cancer therapies.

In a variety of cancers, including CRC, deregulation of AKT signaling has been linked to tumorigenesis, tumor progression, and drug resistance^3–5^. AKT kinase is activated through a dual phosphorylation mechanism. The kinase 3-phosphoinositide-dependent protein kinase 1 (PDK1), which is transported to the membrane on the basis of its Pleckstrin homology (PH) domain, phosphorylates AKT at Thr308 in the activation loop, thereby leading to partial AKT/PKB activation^6^. Phosphorylation of AKT at Ser473 in the carboxy-terminal hydrophobic motif by the mammalian target of rapamycin complex 2 (mTORC2)^7^ induces full AKT activity. AKT can be negatively regulated by the dephosphorylation of Thr308 by protein phosphatase 2 (PP2A)^8^, the dephosphorylation of Ser473 by the PH-domain leucine-rich-repeat-containing protein phosphatase (PHLPP1/2)^9,10^, and the conversion of PIP3 to PIP2 by PTEN^11^. In CRC, the amplification of receptor tyrosine kinase^12^ as well as the frequent mutations in upstream regulators of AKT such as KRAS (mutated in 30%–40% of CRC cases) and PIK3CA (mutated in 10%–15% of CRC cases) lead to the constitutive activation of AKT signaling^13,14^. Drugs targeting AKT have been shown to induce apoptosis and suppress tumor growth^15^, whereas the downregulation of AKT phosphatase PHLPP1 is associated with chemoresistance in CRC^16^, thus indicating that AKT is an important therapeutic target in CRC treatment.

Heat shock protein 47 (HSP47), encoded by the SERPINH1 gene, is a 47-kDa collagen-binding HSP, which belongs to the serine protease inhibitor (serpin) superfamily. HSP47 is the only known substrate-specific endoplasmic reticulum (ER) chaperone, which transiently binds newly synthesized procollagens in the ER and facilitates their maturation and secretion^17^. The role of HSP47 in cancer is largely unexplored, and its functions appear to be inconsistent. For example, HSP47 has been reported to be upregulated in several cancers^18–20^, and elevated HSP47 expression has been associated with poor prognosis^21^. Suppression of HSP47 expression with short interfering RNAs has been shown to significantly inhibit cell proliferation, migration, and invasion in cervical cancer^22^. In breast cancer, the expression of HSP47 is activated during cancer development and progression, and its silencing results in the inhibition of tumor growth *in vivo*^23^. In contrast, in neuroblastoma, there are relatively lower levels of HSP47 expression, partly because of aberrant methylation of promoter CpG islands^24^. Therefore, the molecular mechanism through which HSP47 exerts its function remains unclear and appears to be tissue dependent.

In this study, we demonstrate that HSP47 is up-regulated in CRC, and its expression promotes chemoresistance. We further demonstrate that HSP47 enhances AKT signaling by decreasing the protein stability of PHLPP1, thus suggesting that HSP47 may be a novel therapeutic target for CRC treatment.

## Materials and methods

### Patient cohorts and data analysis

**Figure 1:**
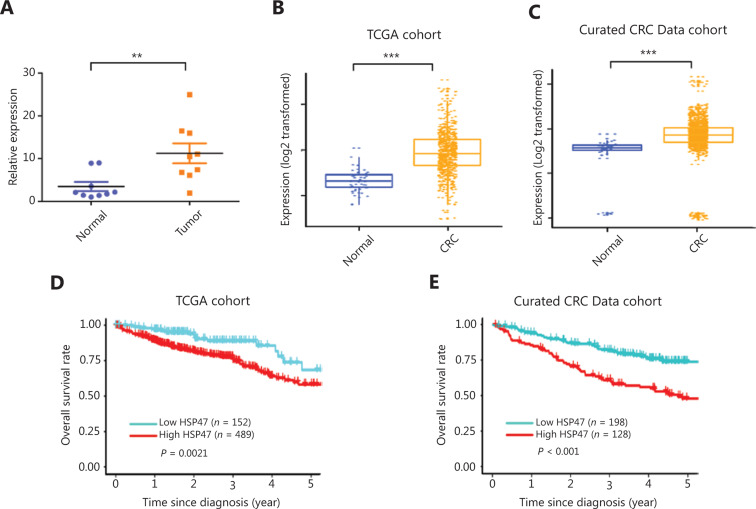
High HSP47 expression in patients with colorectal cancer (CRC) is associated with poor clinical outcomes. (A) Real-time quantitative reverse transcription polymerase chain reaction (qRT-PCR) analysis of HSP47 mRNA expression in paired tumor and adjacent normal tissues collected by laser microdissection from patients with CRC (*n* = 9 pairs) (*P* < 0.01). (B) HSP47 mRNA expression in 644 patients with CRC compared with 51 control tissues in a TCGA cohort. (C) HSP47 mRNA expression in 3,296 patients with CRC compared with 76 control tissues from 22 microarray databases (curated CRC Data). Kaplan–Meier survival analysis of the CRC patients in the (D) TCGA and (E) curated CRC Data cohorts. The patients were divided into high- and low-expression groups on the basis of the cutoff value derived from the receiver operating characteristic (ROC) analysis. **P < 0.01; ***P < 0.001.

Gene expression (RNA-Seq) data and corresponding clinical data from CRC samples were retrieved from two sources: The Cancer Genome Atlas (TCGA) database (http://genome-cancer.ucsc.edu/) and curated CRC Data (http://bioconductor.org/packages/curatedCRCData). Samples included in the study had pathology-confirmed adenocarcinoma of the colon or rectum, were chemotherapy naive, and had available survival information. The survival data were right-censored at 5 years to minimize non-tumor-related causes of death. September 10, 2018 was the date used to calculate overall survival in the TCGA database and curated CRC Data. In addition, CRC tissue samples (and adjacent normal colon tissues) used for real time quantitative reverse transcription polymerase chain reaction (qRT-PCR) analysis (**Figure 1A**) were collected with the approval of the Human Ethics Committee (protocol H14-02808) at the University of British Columbia.

For Kaplan–Meier survival curve analysis, we selected the cutoff scores according to receiver operating characteristic (ROC) curve analysis^25^. The highest score with maximum sensitivity and specificity on the curve was selected as the cutoff point. The data were dichotomized into groups with high and low HSP47 expression, and Kaplan–Meier survival analysis was then conducted^26^. For ROC curve analysis, the clinical outcome and the normalized mRNA expression level index (the number of gene transcripts normalized to the total transcript number from the patient) were dichotomized (dead or alive) in the follow-up data for clinical outcome, high expression and low expression in the index. A log-rank test was used to compare the differences between two groups. ROC curves and Kaplan–Meier survival curves were analyzed with the Survminer package version 0.4.2 in the R version 3.4.1 environment.

The protein expression of HSP47 was examined on the basis of data from The Human Protein Atlas. The data can be downloaded through the following link: https://www.proteinatlas.org/about/download.

### Cell lines and plasmids

The human CRC cell lines RKO, HCT116, and CCL228 were purchased from the American Type Culture Collection (Manassas, VA, USA) and maintained in Dulbecco’s modified Eagle’s medium (Invitrogen, CA, USA) supplemented with 10% newborn calf serum (NCS) at 37 °C in a humidified atmosphere with 5% CO_2_. Resistant cells RKO/5FU, RKO/CPT, HCT116/5FU, and HCT116/CPT were maintained with 100 μM 5-FU, 70 μM CPT-11, 1.25 μM 5-FU, and 1.25 μM CPT-11, respectively. For the establishment of RKO/si-ctrl and RKO/si-HSP47 stable cell lines, RKO cells were transfected with pBAsi-NC (control) or pBAsi-HSP47 plasmids (a gift kindly provided by Dr. Takehiro Kobayashi^27^) with Lipofectamine 2000 (Invitrogen), and this was followed by puromycin (1.5 μg/mL) selection (Thermo Fisher Scientific, Waltham, MA, USA). The non-targeting scrambled control shRNA and pGFP-C-sh-HSP47-A plasmids were purchased from OriGene (Rockville, MD, USA) and were used for transient knockdown experiments. For the construction of a lentiviral vector for expression of HSP47, the full-length human HSP47 gene was prepared by PCR amplification of pZeoSV2 (-)-HSP47 plasmid (a gift kindly provided by Dr. Takehiro Kobayashi^27^) and cloned into the *Bam*HI/*Sal*I sites of pLenti-CMV-GFP-Hygro (Addgene, Cambridge, MA, USA) with the GFP gene removed by restriction enzyme digestion, thus yielding pLenti-CMV-HSP47-Hygro. To establish HCT116/GFP and HCT116/HSP47 stable cell lines, we selected HCT116 cells with hygromycin B (100 μg/mL) (Thermo Fisher Scientific) after viral infection (details below).

### Lentiviral infection

For viral packaging, pGFP-C-shLenti, pGFP-C-shHSP47-A, pLenti-CMV-GFP-Hygro, or pLenti-CMV-HSP47-Hygro was co-transfected at 1:1 mass ratio with a packaging plasmid mix (pCMV-delta R8.74, pRSV-Rev, pCMV-VSV-G, mass ratio 4:1:1) into 80% confluent 293T cells (seeded the day before) with TransIT-Lenti transfection reagent (Mirus, Madison, WI, USA). The virus-containing medium was collected, centrifuged to remove dead cells, and filtered with 0.45 μm filters. The lentiviral supernatants were then concentrated with PEG-it virus precipitation solution (System Biosciences, Palo Alto, CA, USA) and stored at −80 °C in aliquots. For viral infection, the concentrated virus was overlaid onto the indicated cell lines in the presence of polybrene (8 μg/mL) for 48 h, and cell viability assays were then conducted.

### qRT-PCR

Cells were seeded in 60 mm dishes and harvested at 80% confluency. Total RNA was extracted with TRIzol (Invitrogen, Carlsbad, CA, USA) according to the manufacturer’s instructions. Oligo(dT)-primed first-strand cDNA was synthesized from 1 μg RNA with RevertAid H Minus Reverse Transcriptase (Thermo Fisher Scientific) according to the manufacturer’s instructions. The following primers were used for qRT-PCR: RPL19: 5′-TGAAATCGCCAATGCCAACTC-3′ (sense) and 5′-GGCTGTACCCTTCCGCTTACC-3′ (antisense); HSP47: 5′-CTGCAGTCCATCAACGAGTG-3′ (sense) and 5′-CAGCTTTTCCTTCTCGTCGT-3′ (antisense). The transcript levels of RPL19 were used for normalization. qRT-PCR was performed with EvaGreen qPCR Mastermix (Applied Biological Materials, Richmond, BC, Canada) in an ABI Prism 7900HT Sequence Detection System (Applied Biosystems, Foster City, CA, USA). For HSP47 expression analysis of clinical samples (**Figure 1A**), RNA was extracted with an RNeasy FFPE kit (Qiagen, Hilden, Germany) from paraffin-embedded CRC and normal colon tissues. Colonic epithelial cells were identified and captured by laser capture microdissection (μCut Laser Microdissection; Molecular Machine & Industries, Eching, Germany). Five hundred nanograms of RNA from each sample was used for qRT-PCR analysis with the protocol described above.

### Cell viability assays

Cells were seeded at a density of 2,000–3,000 cells per well in 96-well plates. After 24 h, cells were treated with 5-FU at the indicated concentrations for 48 h, and MTS (3-(4,5-dimethylthiazol-2-yl)-5-(3-carboxymethoxyphenyl)-2-(4-sulfophenyl)-2H-tetrazolium, inner salt) assays (Promega, Madison, WI, USA) were then performed according to the manufacturer’s instructions.

### TUNEL assays

Cells were seeded in 4-well chamber slides (Sigma-Aldrich, St. Louis, MO, USA) and treated with 5-FU 24 h post-seeding. Apoptotic cells were detected with a DeadEnd Fluorometric TUNEL System (Promega, WI, USA) according to the manufacturer’s instructions.

### Immunoblotting

Immunoblotting was carried out as previously described^28^. Primary antibodies used in this study are listed below. The anti-HSP47 antibody was obtained from Abcam Biotechnology Company (Cambridge, UK) (ab109117). The anti-PHLPP1 antibody was from Santa Cruz Biotechnology (MO, USA) (sc-390129). The following antibodies were from Cell Signaling (Danvers, MA, USA): rabbit antibodies against AKT (detect total AKT, Cat. 4685), phospho-AKT (S473) (against the phosphorylated residue in all three AKT isoforms, Cat. 9271), and mTOR (Cat. 2972), and phospho-mTOR (S2481) (Cat. 2974). All antibodies were used at a 1:1000 dilution except for the antibody against PHLPP1 (1:200).

### Immunoprecipitation

Cells were harvested in a cold room and fixed with 0.8% paraformaldehyde/phosphate-buffered saline (PBS) at room temperature (RT) for 20 min and 130 mM glycine at RT for 5 min before being lysed in immunoprecipitation (IP) lysis buffer (50 mM HEPES-KOH, pH 7.5, 140 mM NaCl, 5 mM NaF, 2 mM CaCl_2_, 10% glycerol, 0.5% NP-40 and 0.25% Triton X-100) supplemented with proteinase inhibitor (Sigma-Aldrich). Cell lysates were pre-cleared with normal mouse IgG (Sigma) and protein-L beads overnight at 4 °C before incubation with primary antibodies. One hundred micrograms of cell lysates was incubated with 3 μg anti-HSP47 antibody (sc-13150, Santa Cruz Technology) or anti-PHLPP1 antibody (sc-390129, Santa Cruz Technology) overnight at 4 °C, and then incubated with 60 μL protein-L beads (sc-2336, Santa Cruz Technology) overnight at 4 °C. Beads were pulled down and washed in IP lysis buffer according to the manufacturer’s instructions. Proteins were eluted in SDS sample buffer and subjected to Western blot analysis.

### Animal studies

Tumor xenografts of CRC cells (3 × 10^6^ HCT116/GFP and HCT116/HSP47 cells) were resuspended in 100 μL 1:1 PBS/Matrigel (BD Biosciences, Franklin Lakes, NJ, USA) and injected subcutaneously into the right flanks of 8-week-old female nude athymic mice (Simonsen Laboratories, Gilroy, CA, USA) with a 26½ gauge needle. Eight days after implantation, for all measurable tumors > 28.9 mm^3^, treatment was initiated with either PBS or 25 mg/kg 5-FU by intraperitoneal injection. After a 6 day treatment-free period, mice were treated with 5 mg/kg 5-FU every other day until the termination of the study. Tumor size was monitored by measurement of the length and width with calipers, and volumes were calculated with the formula (*L* × *W*^2^) × 0.5, where *L* is the length, and *W* is the width of each tumor. Tumors harvested from the mice were washed twice with sterile PBS and either snap-frozen in liquid nitrogen or fixed with formaldehyde and paraffin embedded (FFPE) for immunofluorescence analysis. Proteins were extracted from the frozen tumor tissues with a TissueLyser (Qiagen, Hilden, Germany). All studies were approved by the Animal Care Committee at the University of British Columbia, Canada (protocol A16-0092).

### Immunofluorescence analysis

Cells were fixed with 4% paraformaldehyde at RT for 15 min, then incubated with blocking buffer (PBS with 5% NCS and 0.2% Triton X-100) for 1 h at RT and with primary antibodies at 4 °C overnight. The proteins were detected with Alexa 647 goat anti-mouse IgG or Alexa 555-goat anti-rabbit IgG (Invitrogen) for 1 h at RT, and this was followed by nuclear staining with Hoechst 33258. Images were captured on a Leica TCS SP5 II confocal microscope (Leica Microsystems, Wetzlar, Germany) with 100× oil objective lenses and a numeric aperture of 1.40 N. Images of the cells were acquired from a 0.13 μm optical section, and no labeling was observed when the secondary antibody was used alone. For the tumor tissue staining, 5 μm slices of the FFPE tissue sections were deparaffinized in xylene and rehydrated through graded ethanol. The sections were incubated with citrate buffer (pH 6.0) in a 95 °C water bath for 20 min for antigen retrieval and then subjected to immunofluorescence staining as described above.

## Results

### Elevated HSP47 expression in the tumors of patients with CRC

Given the variable expression of HSP47 in different cancers, we began by examining the expression of HSP47 in tumor tissues from individuals with CRC. To avoid contamination from areas adjacent to the tumors, we performed laser microdissection to ensure the purity of tumor cells and their paired adjacent normal cells (*n* = 9 pairs) before HSP47 mRNA expression analysis with qRT-PCR. HSP47 was significantly more highly expressed in the CRC tumors than adjacent normal tissues (*P* < 0.01) (**Figure 1A**).

We also analyzed HSP47 expression in the TCGA database and found that it was markedly upregulated in CRC tumors (*P* < 0.001) (**Figure 1B**). A similar result was also obtained from analysis of data compiled from 22 independent microarray databases (curated CRC Data, version 2.12.0) (*P* < 0.001) (**Figure 1C**)^29^. In agreement with the results of this analysis, the protein expression profiles found in The Human Protein Atlas also revealed that HSP47 was upregulated in most CRC tissues (high/medium staining in 8/12 CRC tissues)^30^.

We wondered whether the level of HSP47 expression in CRC might be associated with specific clinical parameters, including survival. Individuals with CRC in either the TCGA or the curated CRC data cohort were grouped into low-HSP47 expression or high-HSP47 expression subgroups according to the optimal cutoff value determined by the ROC curve based on HSP47 expression levels. No association was observed between HSP47 expression and age, sex, race, tumor stage, or tumor site in both cohorts (**Supplementary Table S1 and S2**). However, the log-rank test demonstrated that the 5-year overall survival (OS) for patients with low HSP47 expression in tumor tissues was significantly higher than that in the high-HSP47 group in both the TCGA [hazard ratio (HR) = 0.50 (95% confidence interval, CI 0.33–0.76),* P* = 0.002] (**Figure 1D**) and curated CRC Data cohorts [HR = 2.91 (95% CI 1.92–4.41), *P* < 0.001] (**Figure 1E**). Specifically, individuals with CRC expressing low levels of HSP47 survived 4 months and 23 days longer than those with high HSP47 expression. These results indicate that the expression of HSP47, when upregulated in CRC, may adversely influence tumor response to drug treatment.

### HSP47 expression promotes the resistance of CRC cells to chemotherapy

**Figure 2:**
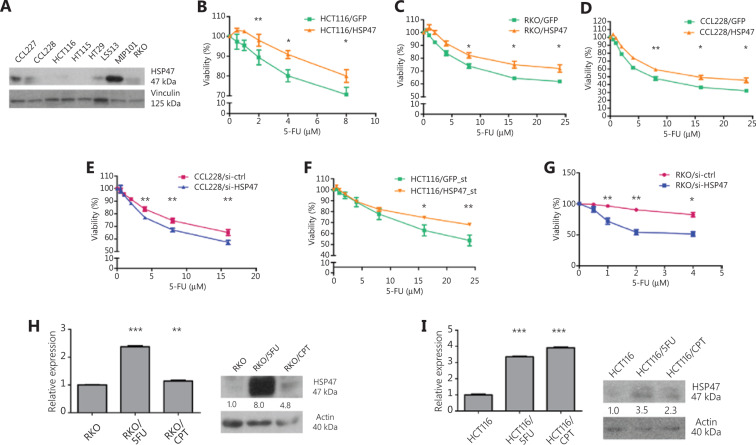
HSP47 expression promotes drug resistance in colorectal cancer (CRC) cells exposed to chemotherapy. (A) Representative image of Western blot analysis of HSP47 protein expression in various human CRC cell lines. (B) HCT116, (C) RKO, and (D) CCL228 CRC cell lines were transiently transduced with HSP47-expression vector and then exposed to 5-FU. MTS cell viability assays of (E) CCL228 cells exposed to various concentrations of 5-FU after HSP47 transient knockdown. MTS assays of (F) HSP47-overexpressing (HCT116/HSP47_st) and (G) HSP47-knockdown (RKO/si-HSP47_st) stable cell lines exposed to 5-FU. HSP47 mRNA and protein expression in (H) RKO and (I) HCT116 resistant cell lines were determined by real-time quantitative reverse transcription polymerase chain reaction (qRT-PCR) and Western blot analysis, respectively. Data represent mean ± SEM, *n* = 3. *P < 0.05; **P < 0.01; ***P < 0.001

To investigate whether HSP47 might influence the response of CRC cells to chemotherapy, we began by examining the viability of CRC cells after the modulation of HSP47 expression level and drug exposure. We selected 3 p53-wild type CRC cell lines (HCT116, CCL228, and RKO) on the basis of their HSP47 expression levels determined by Western blot analysis (**Figure 2A**). After transient transduction with HSP47-overexpression or knockdown lentiviral vectors (**Supplementary Figure S1**), we exposed the 3 CRC cell lines to various concentrations of 5-FU and performed MTS assays. Higher HSP47 expression decreased the sensitivity of CRC cells despite the increasing concentrations of 5-FU. For example, we observed a 10.8 ± 2.1% increase in cell viability after 5-FU (4 μM) exposure in HCT116 cells overexpressing HSP47 (**Figure 2B**) and a 10.1 ± 3.0% and 13.3 ± 3.6% increase in cell viability after 5-FU (24 μM) treatment in HSP47-overexpressing RKO and CCL228 cells, respectively (**Figure 2C, 2D**). In contrast, decreased HSP47 expression increased the sensitivity of CCL228 cells to 5-FU treatment. We observed a 7.8 ± 1.7% decrease in cell viability after 5-FU (16 μM) treatment in the HSP47-knockdown CCL228 cells (**Figure 2E**). We also established cell lines with stable HSP47 overexpression (HCT116/HSP47_st) and knockdown (RKO/si-HSP47_st) and performed downstream analysis. MTS assays on the these stable cell lines demonstrated results consistent with those obtained with transiently expressed cell lines: an increase in expression of HSP47 decreased the sensitivity of CRC cells exposed to 5-FU, whereas HSP47 knockdown had the opposite effect. In the HCT116/HSP47_st and RKO/si-HSP47_st cell lines, 14.4 ± 3.0% higher and 18.8 ± 5.8% lower cell viability were observed after 24 μM and 8 μM5-FU treatment, respectively, in comparison to the levels in the respective control cell lines (**Figure 2F, 2G**). Furthermore, in RKO and HCT116 cell lines with established resistance to 5-FU and CPT-11 (**Figure 2H, 2I**)^31^ the basal levels of HSP47 mRNA and protein expression determined by qRT-PCR and Western blot analysis, respectively, were significantly higher than those in the sensitive parental cell line.

Together, these data suggest that HSP47 may influence the response of CRC cells to chemotherapy.

### HSP47 inhibits apoptosis in CRC cells exposed to chemotherapy

**Figure 3:**
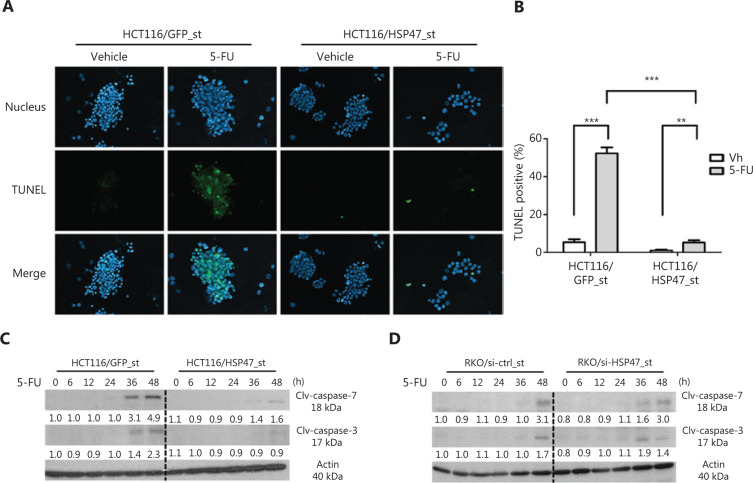
HSP47 inhibits apoptosis in colorectal cancer (CRC) cells exposed to chemotherapy. (A) HCT116/HSP47_st cells were visualized by fluorescence microscopy (200×) 48 h after 50 μM 5-FU treatment. TUNEL-positive nuclei are shown in green, and total nuclei stained with Hoechst 33258 are shown in blue. (B) Quantitative analysis (percentage of TUNEL positive cells *vs.* total) was performed for randomly selected fields (*n* = 10). Representative Western blot images of caspase-3 and -7 cleavage of (C) HCT116/HSP47_st, (D) RKO/si-HSP47_st, and their corresponding control cell lines after treatment with 50 μM 5-FU. Data represent mean ± SEM. **P < 0.01; ***P < 0.001.

Because apoptosis partly influences the clinical response to drug treatment, we further explored the effects of HSP47 on the apoptotic pathway by conducting dUTP-digoxigenin nick end labeling (TUNEL) assays on HSP47-overexpressing HCT116/HSP47_st cells and vector control (HCT116/GFP_st) cells. The percentage of TUNEL-positive cells was significantly lower in HSP47-overexpressing cells (vehicle: 1.0% ± 1.5%; 5-FU: 5.3% ± 3.9%) than in the control cell line (vehicle: 5.3% ± 5.0%; 5-FU: 52.4% ± 9.9%) when the cells were exposed to 5-FU. This result indicated that fewer cells were undergoing apoptosis when cells overexpressed HSP47 (**Figure 3A, 3B**). In line with this observation, there was a similar decrease in the expression of cleaved caspase-3 and -7 in HCT116/HSP47_st cells exposed to 5-FU (**Figure 3C**). Consistent results were also observed in the HSP47 stable-knockdown RKO cells (RKO/si-HSP47_st): cleaved caspase-3 and -7 were detected at earlier time points after exposure to 5-FU (**Figure 3D**). These results suggest that the expression of HSP47 may inhibit the apoptotic pathway, thereby contributing to drug resistance in CRC cells in response to chemotherapy.

### HSP47 promotes the phosphorylation of AKT at Ser473 in CRC cells

**Figure 4:**
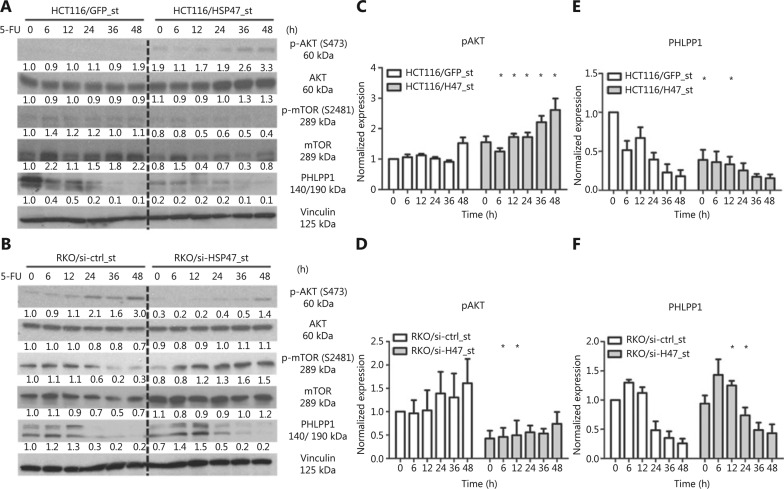
HSP47 promotes AKT activation and decreases PHLPP1 expression in colorectal cancer (CRC) cells exposed to 5-FU. Representative immunoblotting images of (A) HCT116/HSP47_st, (B) RKO/si-HSP47_st, and their corresponding control cell lines treated with 50 μM 5-FU. The immunoblotting images of (C, D) phospho-Akt (S473) and (E, F) PHLPP1 were quantified in Image J software (*n* = 3). Data represent mean ± SEM. *P < 0.05.

Dysregulation of the AKT/PKB pathway has been demonstrated to substantially contribute to therapy-refractory disease in a variety of cancers, including CRC^32–35^. In addition to investigating the anti-apoptotic effects exerted by HSP47, we were interested in examining the effect of HSP47 on AKT signaling. Western blot analysis following the treatment of HCT116/HSP47_st (**Figure 4A**) and RKO/si-HSP47_st (**Figure 4B**) with 5-FU demonstrated increased phosphorylation of AKT at Ser473 in cells with higher HSP47 expression (**Figure 4C, 4D**). However, when we examined the activation of the AKT kinase mTORC2^7^, we found that changes in expression of p-mTOR-S2481 (an mTORC2 activation marker^36^) did not reflect the phosphorylation level of AKT at Ser473. Instead, we observed an elevated expression of the AKT-Ser473 phosphatase PHLPP1^9^ that persisted for a longer period of time in the cell lines with decreased HSP47 expression (**Figure 4E, 4F**). These findings suggest that the changes in the phosphorylation levels of AKT-Ser473 in cells with variable expression of HSP47 are likely to involve PHLPP1.

### HSP47 interacts with PHLPP1 and decreases its protein stability

**Figure 5:**
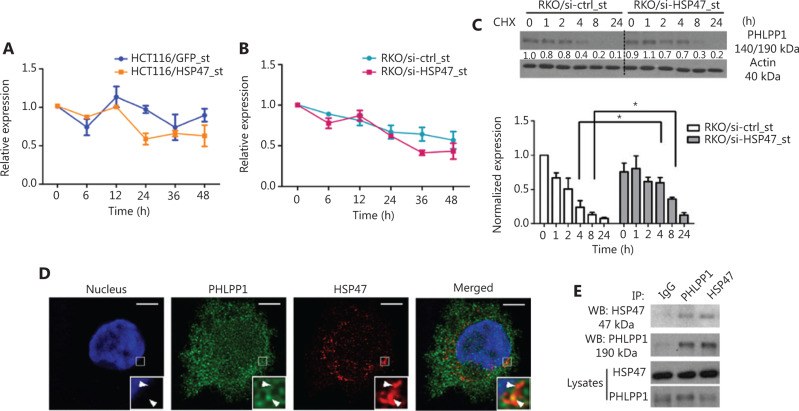
HSP47 interacts with PHLPP1 and decreases its protein stability. Real-time quantitative reverse transcription polymerase chain reaction (qRT-PCR) analysis of PHLPP1 mRNA expression levels in (A) HCT116/HSP47_st, (B) RKO/si-HSP47_st, and their corresponding control cell lines treated with 50 μM 5-FU (*n* = 3). (C) Representative Western blot images of RKO/si-HSP47_st and its control cell line treated with 100 μg/mL cycloheximide (CHX). Cells were collected at the indicated time points and subjected to Western blot analysis. The immunoblotting images were quantified in Image J software (*n* = 3). (D) RKO cells were double stained with the mouse monoclonal antibody against PHLPP1 (green) and rabbit monoclonal antibody against HSP47 (red), then subjected to confocal immunofluorescence analysis. Colocalization between PHLPP1 and HSP47 is indicated by arrows. (E) Co-immunoprecipitation of HSP47 with PHLPP1 in parental RKO cells. Scale bar = 5 μm. Data represent mean ± SEM. *P < 0.05.

To understand how HSP47 influences PHLPP1 expression in CRC, we also examined the mRNA level of PHLPP1 in the cells with qRT-PCR analysis. We observed fluctuations in PHLPP1 mRNA levels in both cell lines during the treatment period; however, the changes in the PHLPP1 mRNA level did not reflect its protein expression patterns during this period, thus suggesting that HSP47 may modulate PHLPP1 expression in a posttranscriptional manner (**Figure 5A, 5B**). To examine this hypothesis, we conducted cycloheximide (CHX) chase assays to determine whether HSP47 might affect the protein stability of PHLPP1. RKO/si-HSP47_st cells were exposed to CHX to suppress further protein synthesis including that of PHLPP1, and PHLPP1 protein was assayed by immunoblotting. As shown in **Figure 5C**, 66.0% ± 8.5% of the PHLPP1 remained in the RKO/si-HSP47_st cells, whereas only 26.7% ± 13.3% of the PHLPP1 was observed in RKO/si-ctrl_st cells after 4 h of CHX exposure, according to the results of densitometry analysis.

Given that HSP47 may influence the stability of PHLPP1, we next examined whether these 2 proteins might interact. Interestingly, immunofluorescence analysis showed that HSP47 and PHLPP1 colocalized in the RKO parental cell line (**Figure 5D**). The interaction between the endogenous HSP47 and PHLPP1 proteins was then validated with co-immunoprecipitation assays (**Figure 5E**). These results demonstrate that HSP47 interacts with PHLPP1, thereby decreasing its protein stability and allowing AKT to remain phosphorylated at Ser473. Consequently, the activation of AKT signaling is prolonged, and cell survival and drug resistance are promoted.

### HSP47 promotes tumor survival *via* AKT activation *in vivo*

**Figure 6:**
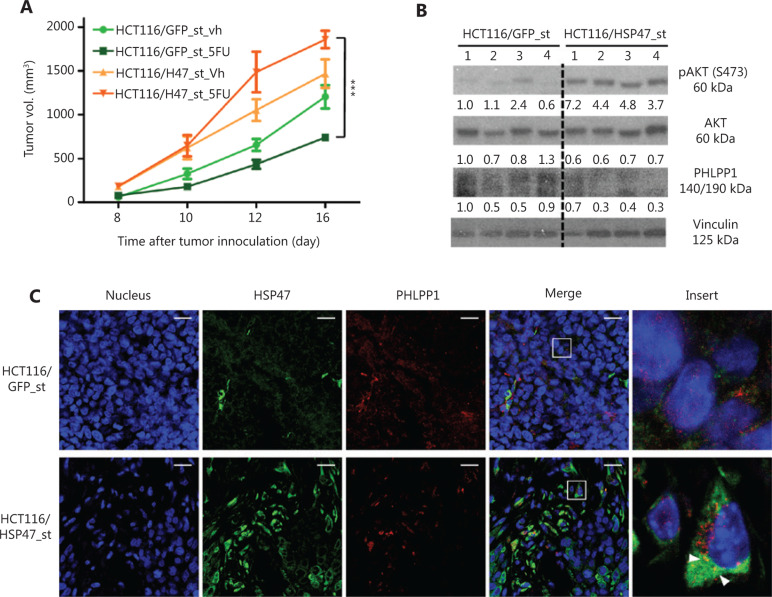
HSP47-overexpressing colorectal cancer (CRC) tumors do not respond to chemotherapy. (A) *In vivo* growth of subcutaneous mouse xenografted tumors of HCT116/HSP47_st and the control cell line treated with either vehicle (Vh) or 5-FU (*n* = 4 per group). (B) Western blot analysis of proteins extracted from tumor tissues collected from vehicle-treated mice. (C) FFPE xenograft tissues were double stained with mouse monoclonal antibody against PHLPP1 (red) and rabbit monoclonal antibody against HSP47 (green), then subjected to confocal immunofluorescence analysis. Arrows indicate colocalization between PHLPP1 and HSP47. Scale bar = 20 μm. Data represent mean ± SEM. ***P < 0.001.

To investigate whether HSP47 might influence tumor survival and therapy resistance *in vivo*, we subcutaneously injected stably HSP47-overexpressing HCT116/HSP47_st and control cells into nude mice. The mice were initially treated with 5-FU at 25 mg/kg, and then treated 6 days later with 5-FU at 5 mg/kg every second day. At 16 days after implantation (8 days after initiation of treatment), the average tumor volume of HCT116/HSP47-bearing mice treated with 5-FU was significantly larger than that in mice injected with the control cell lines exposed to 5-FU (control: 741.9 ± 36.4; HSP47: 1858.3 ± 99.0 (mm^3^), *n* = 4 per group, *P* < 0.001) (**Figure 6A**). Importantly, Western blot analysis of the proteins extracted from the tumor tissues showed higher p-AKT (Ser473) expression in HSP47-overexpressing tumors, whereas the PHLPP1 expression was lower in the same tumors (**Figure 6B**). In addition, immunofluorescence analysis of FFPE xenograft tissues also demonstrated colocalization between HSP47 and PHLPP1 (**Figure 6C**). These results support our *in vitro* findings that HSP47 promotes the survival of CRC tumors and drug resistance after exposure to chemotherapy *in vivo*, by enhancing AKT signaling, probably *via* PHLPP1.

## Discussion

**Figure 7:**
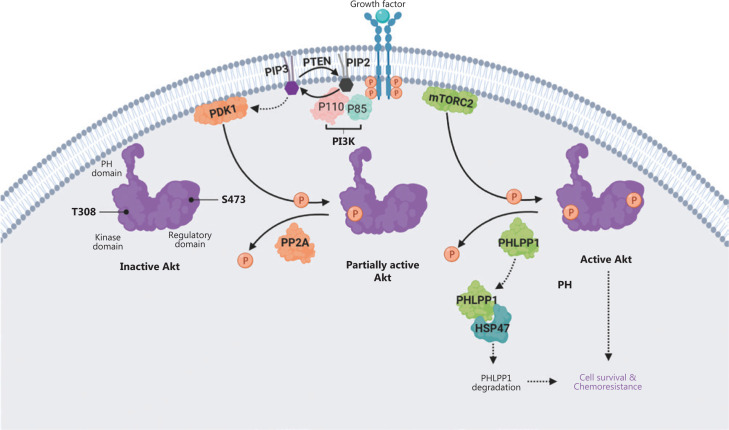
Schematic diagram depicting how HSP47 decreases the protein stability of PHLPP1 and promotes Akt signaling and chemoresistance in colorectal cancer (CRC). The ligand-mediated activation of receptor tyrosine kinase (RTK) promotes recruitment of phosphoinositide 3-kinase (PI3K) to the plasma membrane *via* its regulatory domain (p85), thus triggering activation of PI3K and conversion, *via* the catalytic domain (p110), of phosphatidylinositol-3,4-bisphosphate (PIP2) to phosphatidylinositol-3,4,5-tris-phosphate (PIP3). Akt binds the PIP3 at the plasma membrane *via* its PH domain, thus allowing PDK1 to access and phosphorylate Thr308 in the kinase domain, and leading to partial Akt activation. Phosphorylation of Akt at Ser473 in the regulatory domain by mTORC2 stimulates full Akt activity. Dephosphorylation of Thr308 by PP2A, dephosphorylation of Ser473 by PHLPP1, and the conversion of PIP3 to PIP2 by phosphatase and tensin homolog (PTEN) antagonize Akt signaling. We propose that HSP47 promotes cell survival and chemoresistance by interacting with PHLPP1. The binding of HSP47 facilitates PHLPP1 protein degradation, thereby maintaining and supporting the full activity of Akt kinase required for cell survival in CRC cells receiving chemotherapy.

In this study, we demonstrated that HSP47 promotes cellular survival and decreases drug sensitivity in CRC cells by enhancing the activation of AKT signaling. The expression of HSP47 was significantly elevated in human CRC tissues, and individuals with higher HSP47 expression tended to have poorer overall survival. These findings may be a result of HSP47’s ability to suppresses apoptosis in CRC cells despite exposure to 5-FU. In addition, in HSP47-overexpressing CRC cells, we observed greater and more prolonged activation of AKT when cells were exposed to chemotherapy. In contrast, AKT activity was subdued in knockdown cells with decreased HSP47 expression. Interestingly, the mechanism underlying this persistent activation of AKT in CRC cells with elevated HSP47 expression might be based on this protein’s ability to interact with and destabilize PHLPP1, a phosphatase that dephosphorylates AKT at Ser473. In the presence of decreased PHLPP1 levels and consequently diminished phosphatase activity, AKT retains its phosphorylated state at Ser473, thus promoting cellular survival even in the presence of environmental stressors (such as chemotherapy) (**Figure 7**).

Failure of chemotherapy treatment primarily results from the emergence of drug resistance. Most traditional chemotherapeutic drugs take advantage of one of the main characteristics of cancer cells, their rapid growth rate, by targeting the cell-cycle machinery and inducing DNA damage to inhibit tumor growth. For example, 5-FU is a key agent in first-line chemotherapeutic regimens for CRC therapy. The active metabolites of 5-FU perturb RNA synthesis and inhibit thymidylate synthase, a nucleotide synthetic enzyme, which can be directly misincorporated into DNA, thus causing DNA damage and cell death. The activation of PI3K/AKT/mTOR (PAM) signaling, which inhibits the activation of apoptosis, has been implicated in the resistance to several chemotherapeutic agents, including 5-FU, doxorubicin, and paclitaxel^37,38^. In this study, we demonstrated that HSP47 enhances the resistance of cancer cells toward 5-FU by inhibiting apoptosis activation *via* PAM signaling.

Several ongoing studies are exploring the anti-tumor effects of PAM signaling inhibitors in combination with current regimens^37,39,40^. However, the complicated network of interactions with parallel cascades in PAM signaling allows cancer cells to continue to evade PAM inhibition through negative feedback mechanisms and compensatory signaling pathways. For example, both lung and breast cancer cell lines and patient tumors eventually evade mTOR inhibition after exposure to the rapamycin derivative RAD001 by increasing AKT phosphorylation through the upregulation of IGF-1 signaling^41,42^. Moreover, increased pAKT (Ser473) has also been found in the tumors of patients with CRC and breast cancer after treatment with the mTOR inhibitor everolimus^43^. These studies suggest that instead of targeting mTOR kinase downstream of PAM signaling, direct inhibition of AKT kinase may be advantageous by avoiding a feedback loop that mediates overaction of AKT. However, attempts to develop AKT-specific and isozyme selective inhibitors have also proven difficult, given the high degree of homology in the ATP binding pocket region among AKT, protein kinase A (PKA), and protein kinase C (PKC)^44^. However, in this study, we demonstrate a potential mechanism for targeted AKT inhibition. We show that HSP47 augments AKT signaling in CRC cells *in vitro* and *in vivo*, particularly in the presence of chemotherapy. This effect can be reversed by decreasing HSP47 expression through shRNA knockdown, thus inhibiting AKT as well as a further decline in cell viability in response to chemotherapy. These encouraging findings indicate that HSP47 is a potential novel target for CRC that might be used to inhibit AKT signaling. Moreover, because targeting HSP47 suppresses AKT activity, potentially by providing sustained PHLPP1 phosphatase activity, this strategy may also help to minimize the development of resistance mechanisms, as seen with mTOR inhibitors.

Another benefit of the suppression of AKT activity *via* HSP47 targeting is that, through its interaction with PHLPP1, HSP47 specifically targets AKT2 and AKT3^10^. Although all three isoforms of AKT (AKT1, AKT2, and AKT3) have been reported to be expressed in both normal and CRC tissues^45^, AKT2 is more abundantly overexpressed in late-stage CRC and metastatic tumors, thus suggesting that AKT2 plays a critical role in CRC progression^46^ and may therefore be more susceptible to HSP47 inhibition. Whether HSP47 also interacts with PHLPP1’s isoform PHLPP2, which negatively regulates the activity of AKT1 and AKT3^10^, remains to be explored. If HSP47 is the sole inhibitor of PHLPP1, a potential compensatory response leading to the hyperactivation of AKT1 might be induced in HSP47-targeted therapy, owing to the synergistic and overlapping functions of AKT1 and AKT2 in CRC^47^; this possibility will require further examination.

HSP47 is highly expressed and is associated with abnormal collagen deposition in myofibroblasts and type II pneumocytes in the lungs of individuals with idiopathic pulmonary fibrosis (IPF)^48,49^. Although the role of HSP47 is unclear in IPF, inhibition of HSP47 (with a lipid nanoparticle encapsulating an siRNA against HSP47, ND-L02-s0201) has been shown to improve pulmonary fibrosis in phase I clinical trials^50^. These findings suggest that specific inhibition of HSP47 may be a feasible option for the treatment of CRC.

## Conclusions

We identified HSP47 as a novel protein that promotes cancer survival by modulating AKT signaling, probably *via* PHLPP1, in CRC. HSP47 may serve as a potential therapeutic target in CRC to promote the efficacy of chemotherapy.

## References

[1] Siegel RL, Miller KD, Fedewa SA, Ahnen DJ, Meester RGS, Barzi A (2017). Colorectal cancer statistics, 2017.. CA Cancer J Clin..

[2] Van Cutsem E, Cervantes A, Nordlinger B, Arnold D (2014). Metastatic colorectal cancer: ESMO clinical practice guidelines for diagnosis, treatment and follow-up.. Ann Oncol..

[3] Philp AJ, Campbell IG, Leet C, Vincan E, Rockman SP, Whitehead RH (2001). The phosphatidylinositol 3′-kinase p85alpha gene is an oncogene in human ovarian and colon tumors.. Cancer Res..

[4] Khaleghpour K, Li Y, Banville D, Yu Z, Shen SH (2004). Involvement of the PI 3-kinase signaling pathway in progression of colon adenocarcinoma.. Carcinogenesis..

[5] Zhang J, Roberts TM, Shivdasani RA (2011). Targeting PI3K signaling as a therapeutic approach for colorectal cancer.. Gastroenterology..

[6] Alessi DR, James SR, Downes CP, Holmes AB, Gaffney PR, Reese CB (1997). Characterization of a 3-phosphoinositide-dependent protein kinase which phosphorylates and activates protein kinase Balpha.. Current Biol..

[7] Sarbassov DD, Guertin DA, Ali SM, Sabatini DM (2005). Phosphorylation and regulation of Akt/PKB by the rictor-mTOR complex.. Science..

[8] Andjelkovic M, Jakubowicz T, Cron P, Ming XF, Han JW, Hemmings BA (1996). Activation and phosphorylation of a pleckstrin homology domain containing protein kinase (RAC-PK/PKB) promoted by serum and protein phosphatase inhibitors.. Proc Natl Acad Sci U S A..

[9] Gao T, Furnari F, Newton AC (2005). PHLPP: a phosphatase that directly dephosphorylates Akt, promotes apoptosis, and suppresses tumor growth.. Mol Cell..

[10] Brognard J, Sierecki E, Gao T, Newton AC (2007). PHLPP and a second isoform, PHLPP2, differentially attenuate the amplitude of Akt signaling by regulating distinct Akt isoforms.. Mol Cell..

[11] Stambolic V, Suzuki A, de la Pompa JL, Brothers GM, Mirtsos C, Sasaki T (1998). Negative regulation of PKB/Akt-dependent cell survival by the tumor suppressor PTEN.. Cell..

[12] Ooi A, Takehana T, Li X, Suzuki S, Kunitomo K, Iino H (2004). Protein overexpression and gene amplification of HER-2 and EGFR in colorectal cancers: an immunohistochemical and fluorescent in situ hybridization study.. Modern Pathol..

[13] Wood LD, Parsons DW, Jones S, Lin J, Sjoblom T, Leary RJ (2007). The genomic landscapes of human breast and colorectal cancers.. Science..

[14] Samuels Y, Wang Z, Bardelli A, Silliman N, Ptak J, Szabo S (2004). High frequency of mutations of the PIK3CA gene in human cancers.. Science..

[15] Nakamura Y, Yogosawa S, Izutani Y, Watanabe H, Otsuji E, Sakai T (2009). A combination of indol-3-carbinol and genistein synergistically induces apoptosis in human colon cancer HT-29 cells by inhibiting Akt phosphorylation and progression of autophagy.. Mol Cancer..

[16] Wen YA, Stevens PD, Gasser ML, Andrei R, Gao T (2013). Downregulation of PHLPP expression contributes to hypoxia-induced resistance to chemotherapy in colon cancer cells.. Mol Cell Biol..

[17] Sauk JJ, Nikitakis N, Siavash H (2005). Hsp47 a novel collagen binding serpin chaperone, autoantigen and therapeutic target.. Frontiers Biosci..

[18] Poschmann G, Sitek B, Sipos B, Ulrich A, Wiese S, Stephan C (2009). Identification of proteomic differences between squamous cell carcinoma of the lung and bronchial epithelium.. Mol Cellular Proteomics..

[19] Thierolf M, Hagmann ML, Pfeffer M, Berntenis N, Wild N, Roessler M (2008). Towards a comprehensive proteome of normal and malignant human colon tissue by 2-D-LC-ESI-MS and 2-DE proteomics and identification of S100A12 as potential cancer biomarker.. Proteomics Clin Appl..

[20] Iacobuzio-Donahue CA, Maitra A, Shen-Ong GL, van Heek T, Ashfaq R, Meyer R (2002). Discovery of novel tumor markers of pancreatic cancer using global gene expression technology.. Am J Pathol..

[21] Lee HW, Kwon J, Kang MC, Noh MK, Koh JS, Kim JH (2016). Overexpression of HSP47 in esophageal squamous cell carcinoma: clinical implications and functional analysis.. Dis Esophagus..

[22] Yamamoto N, Kinoshita T, Nohata N, Yoshino H, Itesako T, Fujimura L (2013). Tumor-suppressive microRNA-29a inhibits cancer cell migration and invasion *via* targeting HSP47 in cervical squamous cell carcinoma.. Int J Oncol..

[23] Zhu J, Xiong G, Fu H, Evers BM, Zhou BP, Xu R (2015). Chaperone HSP47 drives malignant growth and invasion by modulating an ECM gene network.. Cancer Res..

[24] Yang Q, Liu S, Tian Y, Hasan C, Kersey D, Salwen HR (2004). Methylation-associated silencing of the heat shock protein 47 gene in human neuroblastoma.. Cancer Res..

[25] Zlobec I, Steele R, Terracciano L, Jass JR, Lugli A (2007). Selecting immunohistochemical cut-off scores for novel biomarkers of progression and survival in colorectal cancer.. J Clin Pathol..

[26] Zhu ZH, Sun BY, Ma Y, Shao JY, Long H, Zhang X (2009). Three immunomarker support vector machines-based prognostic classifiers for stage IB non-small-cell lung cancer.. J Clin Oncol..

[27] Kobayashi T, Uchiyama M (2010). Effect of HSP47 expression levels on heterotrimer formation among type IV collagen alpha3, alpha4 and alpha5 chains.. Biomedical Res (Tokyo, Japan)..

[28] Chern YJ, Wong JCT, Cheng GSW, Yu A, Yin Y, Schaeffer DF (2019). The interaction between SPARC and GRP78 interferes with ER stress signaling and potentiates apoptosis *via* PERK/eIF2alpha and IRE1alpha/XBP-1 in colorectal cancer.. Cell Death Dis..

[29] Parsana P, Riester M, Huttenhower C, Waldron L (2013). CuratedCRCData: Clinically annotated data for the colorectal cancer transcriptome..

[30] Berglund L, Bjorling E, Oksvold P, Fagerberg L, Asplund A, Szigyarto CA (2008). A genecentric human protein atlas for expression profiles based on antibodies.. Mol Cell Proteomics..

[31] Rahman M, Chan AP, Tang M, Tai IT (2011). A peptide of SPARC interferes with the interaction between Caspase8 and Bcl2 to resensitize chemoresistant tumors and enhance their regression in vivo.. PLoS One..

[32] Hafsi S, Pezzino FM, Candido S, Ligresti G, Spandidos DA, Soua Z (2012). Gene alterations in the PI3K/PTEN/AKT pathway as a mechanism of drug-resistance (Review).. Int J Oncol..

[33] Brown KK, Toker A (2015). The phosphoinositide 3-kinase pathway and therapy resistance in cancer.. F1000 Prime Rep..

[34] Morkel M, Riemer P, Blaker H, Sers C (2015). Similar but different: Distinct roles for KRAS and BRAF oncogenes in colorectal cancer development and therapy resistance.. Oncotarget..

[35] Danielsen SA, Eide PW, Nesbakken A, Guren T, Leithe E, Lothe RA (2015). Portrait of the PI3K/AKT pathway in colorectal cancer.. Biochim Biophys Acta..

[36] Copp J, Manning G, Hunter T (2009). TORC-specific phosphorylation of mammalian target of rapamycin (mTOR): phospho-Ser2481 is a marker for intact mTOR signaling complex 2.. Cancer Res..

[37] Hirai H, Sootome H, Nakatsuru Y, Miyama K, Taguchi S, Tsujioka K (2010). MK-2206, an allosteric Akt inhibitor, enhances antitumor efficacy by standard chemotherapeutic agents or molecular targeted drugs in vitro and in vivo.. Mol Cancer Ther..

[38] Burris HA (2013). Overcoming acquired resistance to anticancer therapy: focus on the PI3K/AKT/mTOR pathway.. Cancer Chemother Pharmacol..

[39] Davies BR, Greenwood H, Dudley P, Crafter C, Yu DH, Zhang J (2012). Preclinical pharmacology of AZD5363, an inhibitor of AKT: pharmacodynamics, antitumor activity, and correlation of monotherapy activity with genetic background.. Mol Cancer Ther..

[40] Toren P, Kim S, Cordonnier T, Crafter C, Davies BR, Fazli L (2015). Combination AZD5363 with enzalutamide significantly delays enzalutamide-resistant prostate cancer in preclinical models.. Eur Urol..

[41] Sun SY, Rosenberg LM, Wang X, Zhou Z, Yue P, Fu H (2005). Activation of Akt and eiF4E survival pathways by rapamycin-mediated mammalian target of rapamycin inhibition.. Cancer Res..

[42] O’Reilly KE, Rojo F, She QB, Solit D, Mills GB, Smith D (2006). mTOR inhibition induces upstream receptor tyrosine kinase signaling and activates Akt.. Cancer Res..

[43] Tabernero J, Rojo F, Calvo E, Burris H, Judson I, Hazell K (2008). Dose- and schedule-dependent inhibition of the mammalian target of rapamycin pathway with everolimus: a phase I tumor pharmacodynamic study in patients with advanced solid tumors.. J Clin Oncol..

[44] Rodon J, Dienstmann R, Serra V, Tabernero J (2013). Development of PI3K inhibitors: lessons learned from early clinical trials.. Nat Rev Clin Oncol..

[45] Zinda MJ, Johnson MA, Paul JD, Horn C, Konicek BW, Lu ZH (2001). Akt-1, -2, and -3 are expressed in both normal and tumor tissues of the lung, breast, prostate, and colon.. Clin Cancer Res..

[46] Rychahou PG, Kang J, Gulhati P, Doan HQ, Chen LA, Xiao SY (2008). Akt2 overexpression plays a critical role in the establishment of colorectal cancer metastasis.. Proc Natl Acad Sci U S A..

[47] Ericson K, Gan C, Cheong I, Rago C, Samuels Y, Velculescu VE (2010). Genetic inactivation of AKT1, AKT2, and PDPK1 in human colorectal cancer cells clarifies their roles in tumor growth regulation.. Proc Natl Acad Sci U S A..

[48] Iwashita T, Kadota J, Naito S, Kaida H, Ishimatsu Y, Miyazaki M (2000). Involvement of collagen-binding heat shock protein 47 and procollagen type I synthesis in idiopathic pulmonary fibrosis: contribution of type II pneumocytes to fibrosis.. Human Pathol..

[49] Kakugawa T, Mukae H, Hayashi T, Ishii H, Nakayama S, Sakamoto N (2005). Expression of HSP47 in usual interstitial pneumonia and nonspecific interstitial pneumonia.. Respiratory Res..

[50] Hagiwara S, Iwasaka H, Matsumoto S, Noguchi T (2007). Antisense oligonucleotide inhibition of heat shock protein (HSP) 47 improves bleomycin-induced pulmonary fibrosis in rats.. Respiratory Res..

