# Supplementary material for: Heat shock protein 47 promotes tumor survival and therapy resistance by modulating AKT signaling via PHLPP1 in colorectal cancer
Source: Cancer Biol Med. 2020 May 15;17(2):343–56. doi: 10.20892/j.issn.2095-3941.2019.0261 (PMC7309463; doi:10.20892/j.issn.2095-3941.2019.0261)
Supplement: Supplementary file 1 [file cbm-17-343-s001.pdf]

Supplementary materials

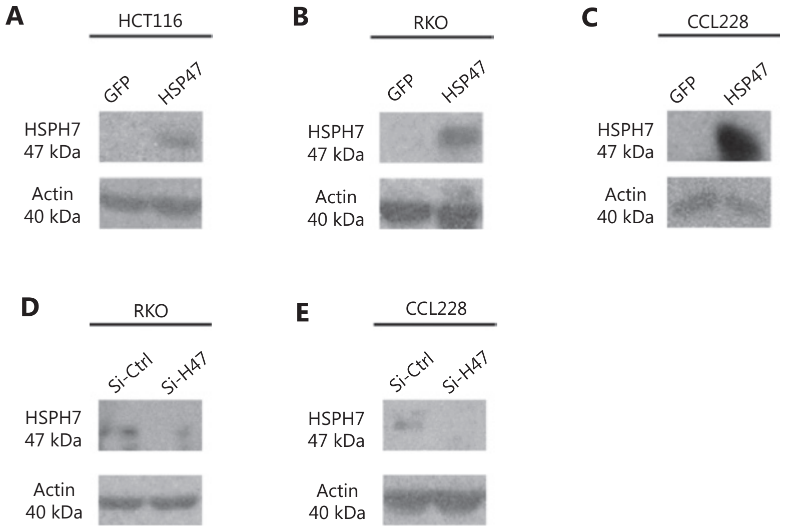

Figure S1 Examination of HSP47 expression level in CRC cell lines with HSP47 transient overexpression or knockdown. Representative images of Western blot analysis of HSP47 expression level in (A) HCT116, (B) RKO cells and (C) CCL228 cell lines after transient transduction of HSP47-overexpressing vectors. Western blot analysis of HSP47 expression level in (D) RKO and (E) CCL228 cells after transient knockdown of HSP47.

Table S1 Association between HSP47 expression and clinical information in the TCGA cohort

Variables	*n*	High HSP47 (total *n* = 489)	Low HSP47 (total *n* = 152)	χ^2	*P*
Gender				0.006	0.940
Male	342	260	82		
Female	299	229	70		
Age (years)				2.594	0.458
< 45	38	32	6		
45–59	139	109	30		
60–80	377	280	97		
> 80	85	66	19		
Race				11.635	0.020
American native	1	1	0		
Black	68	44	24		
White	311	230	81		
Asian	12	11	1		
Not reported	249	203	46		
Tumor stage				6.283	0.099
I	111	77	34		
II	237	177	60		
III	183	149	34		
IV	90	71	19		
Tumor site				3.612	0.307
Ascending	206	146	60		
Transverse	26	18	8		
Descending	133	99	34		
Rectal	163	129	34		

Table S2 Association between HSP47 expression and clinical information in the curated CRC Data cohort

Variables	*n*	High HSP47 (total *n* = 128)	Low HSP47 (total *n* =198)	χ^2	*P*
Gender				0.095	0.758
Male	171	69	102		
Female	155	59	96		
Age				6.852	0.077
< 45	22	13	9		
45–59	84	37	47		
60–80	188	64	124		
> 80	32	14	18		
Race				0.997	0.802
Black	13	8	5		
White	201	105	96		
Hispanic	2	1	1		
Other	16	10	6		
Tumor stage				4.060	0.255
I	42	12	30		
II	100	37	63		
III	105	48	57		
IV	79	31	48		
Tumor site				22.279	2.00E-06
Colon	261	125	136		
Rectal	33	1	32		
